# Impact of valvular heart disease on hip replacement: a retrospective nationwide inpatient sample database study

**DOI:** 10.1186/s12891-021-04738-z

**Published:** 2021-10-09

**Authors:** Qiang Lian, Jian Wang, Yun Lian, Qinfeng Yang, Mingchen Zhao, Yang Zhang

**Affiliations:** 1 Division of Orthopaedic Surgery, Department of Orthopaedics, Nanfang Hospital, Southern Medical University, 1838 Guangzhou Avenue, Guangzhou, 510515 Guangdong China; 2 First Affiliation Hospital of Nanchang University, Nanchang City, Jiangxi Province China; 3 Goodwill Hessian Health Technology Co., Ltd., Beijing, 100007 China

**Keywords:** Valvular heart disease, Hip replacement, Aortic stenosis

## Abstract

**Background:**

To study the impact of valvular heart disease (VHD) on hip replacement, particularly the clinical impactions of aortic stenosis before total/partial hip arthroplasty.

**Methods:**

This was a retrospective cohort study. Data on patients who had undergone hip replacement from 2005 to 2014 were extracted from the NIS database. Independent t test and chi-square test were used to analyze the essential characteristics of patients. Multivariate regression was used to estimate the correlation among demographics, comorbidities, complications, hospitalization costs, and time.

**Results:**

VHD accounted for 5.56% and AS accounted for 0.03% of the patients before hip replacement surgeries. Patients with VHD before hip replacement are related to the following characteristics: female patients (odds ratio [OR] = 1.15 [1.12–1.18]), elective admission (OR = 0.78 [0.76–0.80]), Charlson Comorbidity Index ≥3 (OR = 1.06 [1.03–1.08]), large-volume hospitals (OR = 1.13 [1.1–1.2]), teaching hospitals (OR = 5 4.4 [2.9–6.7]), and hospital location in urban areas (OR = 1.22 [1.2–1.3]). In addition, VHD is a risk factor for mortality and some acute postoperative medical complications, such as acute cardiac event (OR = 2.96 [2.87–3.04]), acute pulmonary edema (OR = 1.13 [1.06–1.21]), acute cerebrovascular event (OR = 1.22 [1.16–1.74]), and acute renal failure (OR = 1.22 [1.17–1.27]). It also has an impact on DVT/PE (OR = 0.89 [0.8–0.99]). Patients with AS before hip replacement have basic demographic characteristics like those of hip replacement patients with valvular disease. Patients with AS are older than those without AS before surgery (OR = 3.28 [2.27–4.75) and are related to the following characteristics: female patients (OR = 1.92 [1.32–2.8]) and elective admission (OR = 0.51 [0.36–0.75]). The perioperative period is limited to acute postoperative complications, such as acute cardiac events (OR = 2.50 [1.76–3.53]) and acute hepatic failure (OR = 7.69 [1.8–32.89]). Both valvular diseases and AS are associated with a higher mortality rate and hospitalization cost.

**Conclusion:**

VHD independently predicted mortality rate and surgical and medical complications after total/partial hip arthroplasty.

**Supplementary Information:**

The online version contains supplementary material available at 10.1186/s12891-021-04738-z.

## Background

Currently, the older adult population of the world is growing at an unprecedented rate [1]. The incidence of osteoporosis, osteoarthritis and femoral neck fractures increases with age. Simultaneously, with the increasing number of people longing for an improved quality of life, hip replacements are becoming more popular among the older adult population [2].

However, the prevalence and severity of valvular diseases sharply increase with age. According to a previous study, people with valvular heart disease (VHD) account for 13.3% of those aged ≥75 years [3, 4]. The valve is an inseparable part of the heart, and its stenosis or insufficiency has a significant impact on heart function. Valvular diseases will gradually develop into heart failure with increasing age. However, unlike coronary artery bypass surgery, the low operation volume of valve replacement and the low mortality attributed to valvular diseases in the USA make people not regard it as a major public health problem [3]. Such a view that the contribution of valvular diseases to mortality and morbidity might be even lower than in the average population could lead to a snowballing effect in clinics. With the improvement in the understanding and treatment of valvular diseases, their impact on the community should be re-evaluated. For patients with VHD undergoing hip replacement surgery, a detailed assessment of postoperative complications in the perioperative period is also needed.

In Western countries, aortic stenosis (AS) remains the most prevalent type of valvular disease. There is clinical evidence showing that the diagnosis of AS gradually increases as the population continues to age. Studies have shown that the severity of AS increases with age and older individuals over the age of 75 years (nearly 10%) have a high prevalence of severe stenosis [5]. AS is an established predictor of perioperative complications following cardiac and noncardiac surgery. Moreover, severe AS is considered a high-risk indicator of cardiac complications during noncardiac surgery; blood loss and tachycardia related to anesthesia and surgery can further lower coronary perfusion and may ultimately lead to myocardial infarction or death.

There are a few pieces of literature that analyze the impact of valvular diseases on hip replacement. In a case-control study by Keswani et al., the effect of AS on hip fracture was investigated, although the number of cases included in the study was small [6]. Another research has shown that heart failure is a risk factor for hip fractures. It may be attribute to the fact that heart failure is related to osteoporosis and other potential common risk factors with hip fracture [7]. Heart failure is also known as the endpoint of most valvular diseases and is the most common complication of AS. Yet few studies analyzed the impact of valvular disease on hip replacement. Furthermore, there are some studies about bridging anticoagulation after a valve replacement. However, a conclusion has not been reached regarding whether it is necessary to treat valvular diseases before hip replacement, particularly for emergency hospital admission patients, such as those having femoral neck fractures. Therefore, understanding the perioperative impact of valvular diseases on patients undergoing hip replacement helps weigh the pros and cons and the sequence of the corresponding valve surgery and hip replacement, as well as prevent postoperative complications after hip replacement. Therefore, it is necessary to explore the impact of valvular diseases on hip replacement.

## Methods

A retrospective analysis was conducted using the 2005–2014 Healthcare Cost and Utilization Project – Nationwide Inpatient Sample (HCUP–NIS), the largest publicly available all-payer inpatient care database in the United States and consisting of approximately 8 million hospital stays each year. The corresponding procedural ICD-9 codes for total and partial hip arthroplasty, which are employed to identify hip replacement patients, are 8151 and 8152, respectively.

According to Ward et al., the definition of VHD included mitral valve disease (394.x, 424.0), aortic valve disease (395.x, 424.1), both mitral and aortic valve disease (396.x), tricuspid or pulmonic valve disease (397.x), other rheumatic heart disease (398.9), and history of valve replacement (V42.2, V43.3) [8]. AS was defined by International Classification of Diseases (ninth revision) Clinical Modification (ICD-9-CM) diagnostic codes including congenital stenosis of aortic valve (746.3), mitral valve insufficiency and aortic valve stenosis (396.2), mitral valve stenosis and aortic valve stenosis (396.0), rheumatic aortic stenosis with insufficiency (395.2), rheumatic aortic stenosis (395.0), aortic valve disorders (424.1). Patients who were less than 18 years of age were excluded [9].

Demographic information, including medical history and comorbidities as well as the length of hospital stay and postoperative complications and mortality, was extracted from the database. All postoperative complications and deaths occurred in the hospital and were recorded in the discharge diagnosis with the corresponding ICD-9-CM. Complications were divided into two parts: acute medical complications and acute surgical complications. The details of the complications are shown in Supplemental Table 1.

Statistical analyses were performed using Stata version 13.1 (StataCorp, LP, College 85 Station, TX, USA). Significant differences between different groups were determined by independent t test for continuous data and chi-square test for categorical data. To identify independent risk factors of VHD, multivariate logistic regression with the stepwise method was performed. All variables, including age groups, races, nature of admission, comorbidity score and postoperative complications were entered into the regression analysis. Multivariate logistic regression models were constructed to assess the association of postoperative complications with VHD or AS after controlling for other elements in the model at the same time. Odds ratios (ORs) and 95% confidence intervals (CIs) were reported by univariate and multivariate analyses. *P*-values < 0.05 with ORs and 95% CIs were used to determine whether independent variables were statistically significant.

## Results

**Fig. 1:**
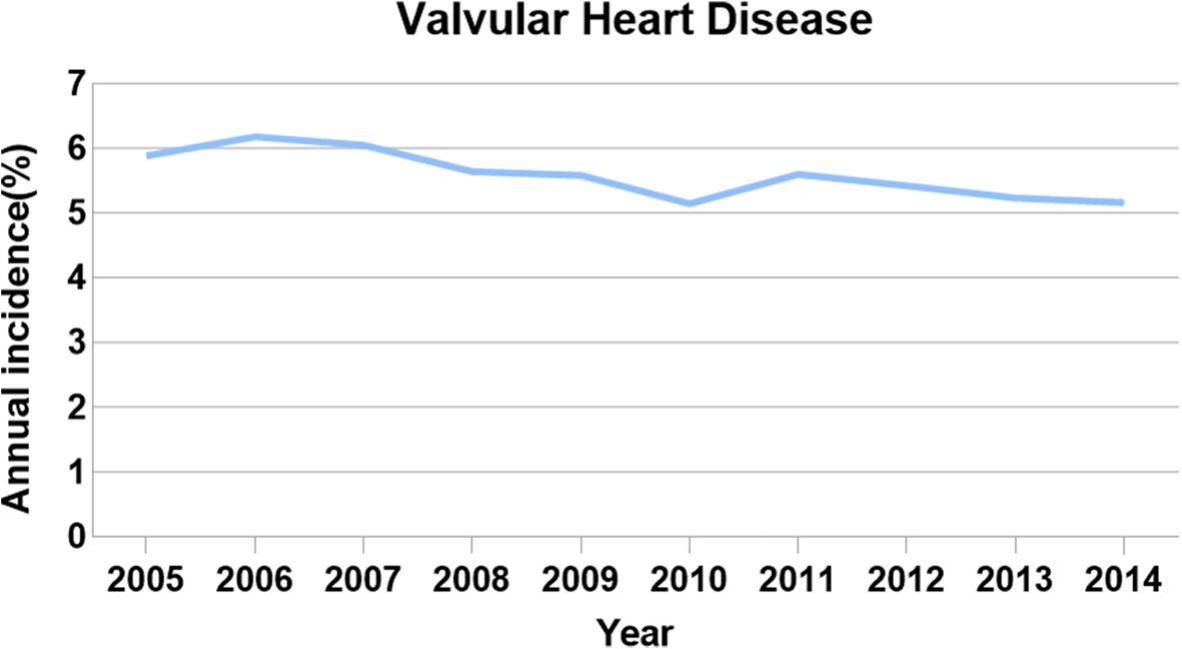
Annual incidence of Valvular Heart Disease undergoing Hip Replacement from 2005 to 2014

**Fig. 2:**
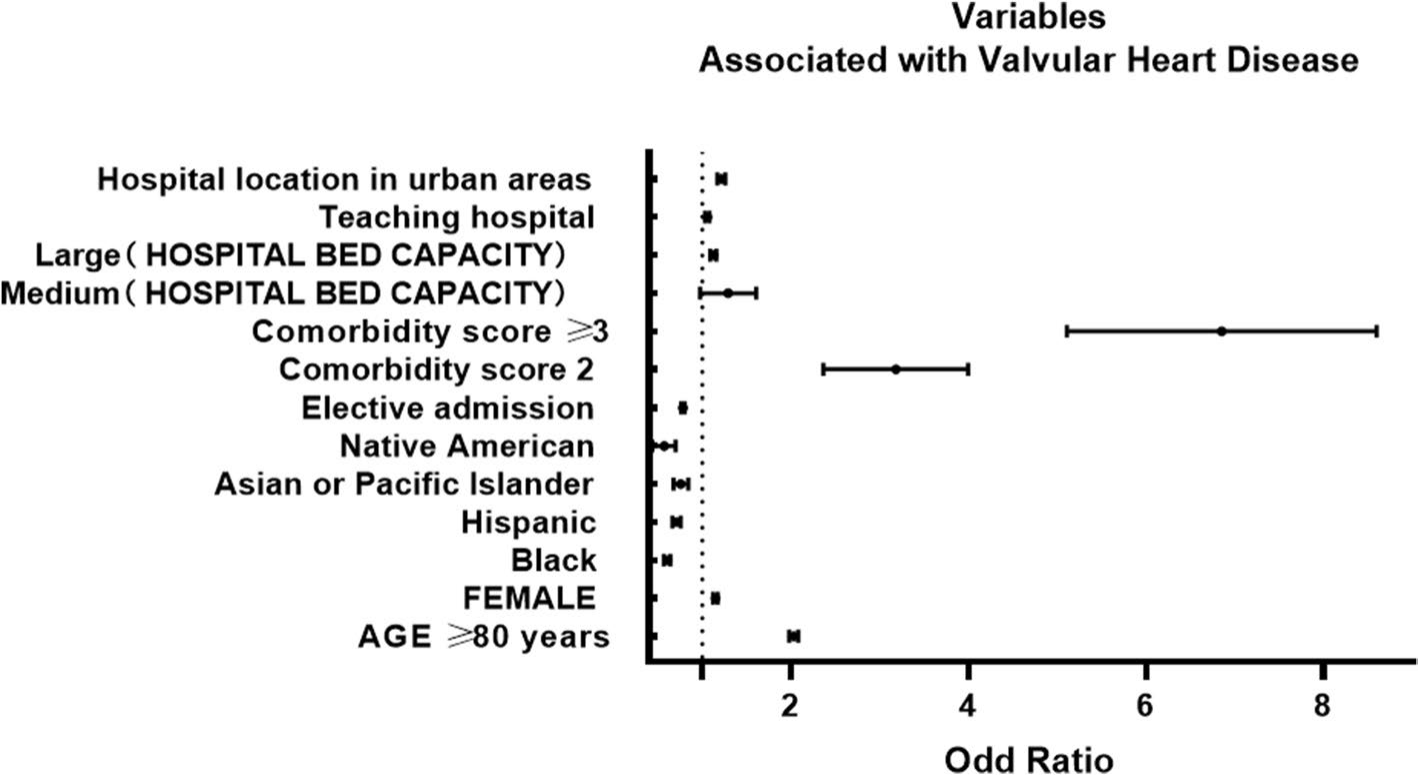
Variables Significantly Associated with Valvular Heart Disease

**Fig. 3:**
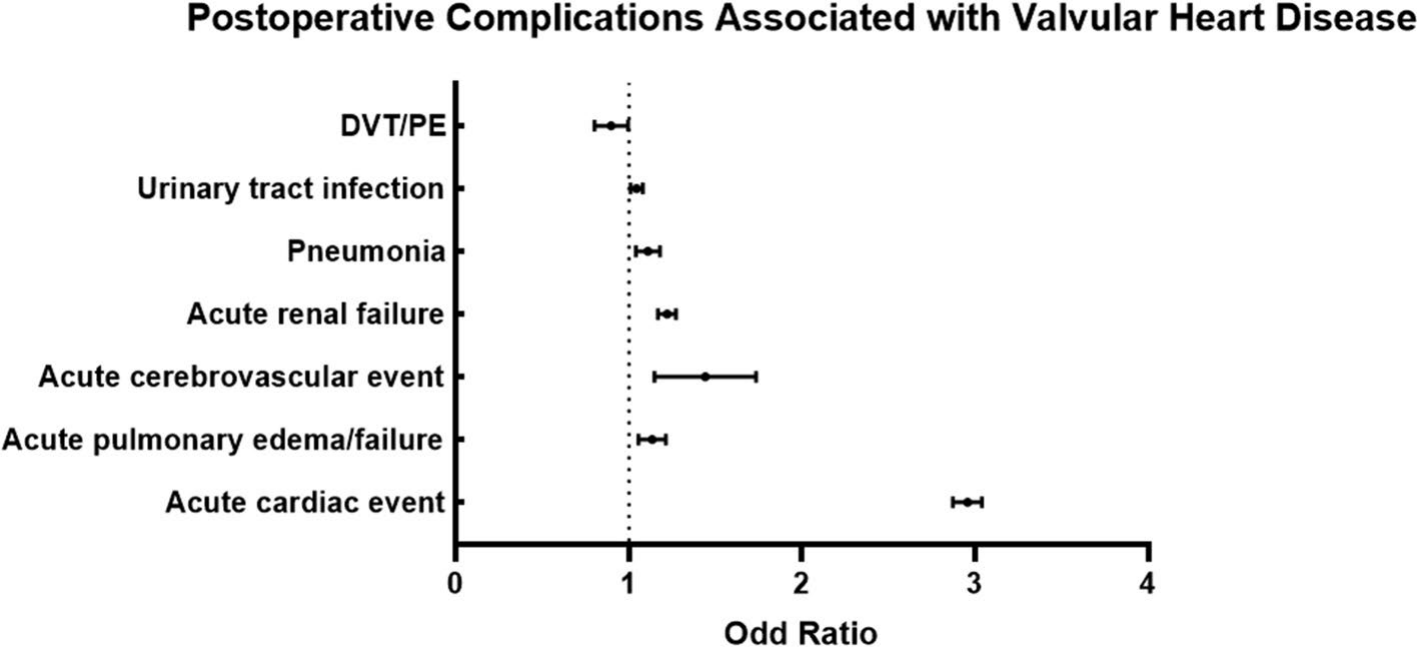
Postoperative Complications Significantly Associated with Valvular Heart Disease

Data on 801,310 cases of hip replacement performed between 2005 and 2014 were collected, of which 44,557 (5.56%) cases had a valvular disease before surgery (Fig. 1). Most of the patients with valvular disease are more likely to be over 80 years of age, female, and have advanced comorbidity. Patients with valvular disease (92.3%) had significantly more common comorbid conditions (comorbidity score, ≥3) than those without valvular disease (74.7%; *P* < 0.0001) (Table 1). Patients with valvular disease were more likely to be urgently admitted and associated with several postoperative medical and surgical complications, such as the mechanical complication of a prosthetic joint (Table 2). Further, the cost of hospitalization and death rate both were higher in patients with valvular disease (Table 2). The multivariate logistic regression analysis of variables associated with patients with valvular disease is shown in Tables 3 and 4. After controlling for the effect of other variables, valvular disease was significantly associated with an increased likelihood of age over 80 years, female sex, white race, high comorbidity score, medium and large hospital bed capacity, a teaching hospital, and a hospital located in an urban area (Fig. 2 and Table 3). In addition, valvular disease was significantly associated with acute cardiac event, acute pulmonary edema/failure, acute cerebrovascular event, acute renal failure (ARF), pneumonia, and urinary tract infection (Fig. 3 and Table 4). VHD was associated with decreased odds of elective admission and DVT/PE (Fig. 3 and Table 4). However, the mechanical complication of a prosthetic joint is not significantly associated with valvular disease.

**Table 1:** Demographic characteristics of included cases

	Heart valvular disease (*N* = 44,557)	No Heart valvular disease (*N* = 756,753)	*P* value
**Incidence rate**	5.56%		< 0.0001
**Age group**			< 0.0001
≤ 40	0.38%	2.44%	
40–64	13.28%	35.31%	
65–80	37.83%	40.22%	
≥ 80	48.52%	22.04%	
**Sex**			< 0.0001
Male	32.50%	40.42%	
Female	67.50%	59.58%	
**Race/ethnicity**			< 0.0001
White	91.27%	86.51%	
Black	3.38%	6.50%	
Hispanic	2.59%	3.56%	
Asian or Pacific Islander	0.93%	1.08%	
Native American	0.21%	0.36%	
Other	1.61%	1.98%	
**Nature of admission**			< 0.0001
Elective admission	48.78%	70.88%	
Non-elective admission	51.22%	29.12%	
**Comorbidity**			< 0.0001
1	0.18%	1.68%	
2	7.54%	23.63%	
≥ 3	92.28%	74.69%

**Table 2:** In-hospital medical characteristics of included cases

	Heart valvular disease (*N* = 44,557)	No Heart valvular disease (*N* = 756,753)	*P* value
**Medical complications**			
Acute cardiac event	22.65%	5.76%	< 0.0001
Acute pulmonary edema/failure	2.93%	1.09%	< 0.0001
Acute cerebrovascular event	0.29%	0.15%	< 0.0001
Acute renal failure	8.01%	3.43%	< 0.0001
Acute hepatic failure	0.14%	0.05%	< 0.0001
Pneumonia	3.48%	1.5%	< 0.0001
Sepsis	0.00%	0.01%	0.7759
Urinary tract infection	12.78%	7.07%	< 0.0001
**Surgical complications**			
Postoperative infection	0.12%	0.09%	0.0800
Non-healing surgical wound	0.02%	0.01%	0.1078
Accidental perforation or laceration of blood vessel, nerve, or organ	0.06%	0.04%	0.3368
Mechanical complication of prosthetic joint	1.55%	1.3%	< 0.0001
DVT/PE	1.01%	0.65%	< 0.0001
**Average cost of hospitalization**	47,578.5$	44,198$	< 0.0001
**Death rate**	2.11%	0.65%	< 0.0001

**Table 3:** Multivariate logistic regression analysis of variables significantly associated with heart valvular disease

Variable	Odds Ratio	95% CI	*P* value
**Age ≥ 80 years**	2.0307	[1.9812,2.0814]	< 0.0001
**Female**	1.1486	[1.1225,1.1753]	< 0.0001
**Race**			
Black	0.604	[0.5702,0.6398]	< 0.0001
Hispanic	0.712	[0.6667,0.7604]	< 0.0001
Asian or Pacific Islander	0.7558	[0.6776,0.843]	< 0.0001
Native American	0.5615	[0.4452,0.7082]	< 0.0001
Other	0.8521	[0.7841,0.9259]	0.0002
**Elective admission**	0.7813	[0.762,0.8011]	< 0.0001
**Comorbidity score 2**	3.1111	[2.4021,4.0293]	< 0.0001
**Comorbidity score ≥ 3**	6.7043	[5.1874,8.6647]	< 0.0001
**Hosp_bedsize**			
Medium	1.0907	[1.6543,1.1283]	< 0.0001
Large	1.1256	[1.0917,1.1606]	< 0.0001
**Teaching hospital**	1.0573	[1.0342,1.0810]	< 0.0001
**Hospital location in Urban**	1.2155	[1.1717,1.2610]	< 0.0001

**Table 4:** Multivariate logistic regression analysis of postoperative complications significantly associated with heart valvular disease

Variable	Odds Ratio	95% CI	*P* value
**Medical complications**			
Acute cardiac event	2.9551	[2.8722,3.0403]	< 0.0001
Acute pulmonary edema/failure	1.1322	[1.0565,1.2134]	0.0004
Acute cerebrovascular event	1.4208	[1.1580,1.7434]	0.0008
Acute renal failure	1.2190	[1.1687,1.2716]	< 0.0001
Acute hepatic failure	0.8647	[0.6379,1.1721]	0.3489
Pneumonia	1.1080	[1.0401,1.1802]	0.0015
Urinary tract infection	1.0439	[1.0094,1.0796]	0.0121
**Surgical complications**			
Mechanical complication of prosthetic joint	0.9595	[0.8808,1.0454]	0.3446
DVT/PE	0.8943	[0.8021,0.9972]	0.0443

In 2005–2014, there were only 204 cases with AS before hip arthroplasty, accounting for approximately 0.03% of the patients before hip replacement surgeries. Most of the general characteristics of patients with AS were similar to those of valvular disease (Tables 5 and 6); On the other hand, acute medical complications were more limited to acute cardiac event and acute hepatic failure (Table 7). The total hospitalization cost is also 13% higher than the nation’s healthcare dollars compared with that of an average patient. Moreover, AS is associated with a higher death rate (Table 5).

**Table 5:** Demographic characteristics

	Aortic stenosis (*N* = 204)	No aortic stenosis (*N* = 801,104)	*P* value
**Incidence rate**	0.03%		
**Age group**			0.0005
≤ 40	0	2.32%	
40–64	3.88%	34.09%	
65–80	26.7%	40.09%	
≥ 80	69.42%	23.5%	
**Sex**			< 0.0001
Female	80.1%	60.02%	
**Race/ethnicity**			< 0.0001
White	89.13%	86.78%	
Black	1.63%	6.32%	
Hispanic	5.98%	3.51%	
Asian or Pacific Islander	2.72%	1.07%	
Native American	0.54	0.36%	
Other	0	1.96%	
**Nature of admission**			< 0.0001
Elective admission	30.58%	69.67%	
**Comorbidity**			0.0005
1	0	1.6%	
2	2.43%	22.74%	
≥ 3	97.57%	75.66%	
**Average cost of hospitalization**	50,131$	44,366$	< 0.0001
**Death rate**	2.43%	0.73%	< 0.0001

**Table 6:** Demographic characteristics

	Aortic stenosis (*N* = 204)	No aortic stenosis (*N* = 801,104)	*P* value
Acute cardiac event	55	53,628	< 0.0001
Acute pulmonary edema/failure	6	9543	0.0381
Acute renal failure	18	29,517	0.0002
Acute hepatic failure	3	475	0.0003
Pneumonia	6	12,869	0.1522
Urinary tract infection	39	59,193	< 0.0001

**Table 7:** Multivariate logistic regression analysis of postoperative complications significantly associated with aortic stenosis

Variable	Odds Ratio	95% CI	*P* value
Acute cardiac event	2.4984	[1.7643,3.5379]	< 0.0001
Acute pulmonary edema/failure	0.6691	[0.2392,1.8715]	0.4431
Acute renal failure	1.0104	[0.5859,1.7425]	0.9702
Acute hepatic failure	7.6935	[1.7994,32.8938]	0.0059
Pneumonia	0.9303	[0.2392,1.8715]	0.866
Urinary tract infection	1.1782	[0.8031,1.7285]	0.4017

## Discussion

In this study, valvular disease was significantly related to high hospitalization expenses and severe complications, such as high hospital death rate, postoperative acute medical diseases, and venous thrombosis. Compared with other valvular diseases, AS is positively associated with high death rates and high hospitalization expenses. However, it is only related to several acute medical complications, in contrast to other valvular diseases. In addition, there were only 204 cases of AS in the more than 800,000 hip replacement cases during 2005–2014, of which 165 were female patients. Most of the cases were not from an elective admission. Fifty-five cases had acute cardiac complications. There were 18, 3, and 39 cases of ARF, liver failure, and postoperative urinary tract infection, respectively. In contrast to the results of a study conducted in 2008, no adverse complications were observed in the 22 cases of joint replacement with AS during 1994–2005 [10].

In one study among the older adult population over the age of 75 years, the proportion of severe AS cases was 3.4%, and the population prevalence rate was 12%. Another study found that AS exponentially increases with age: 3.9 and 9.8% in those aged 70–79 years and 80–89 years, respectively [11]. However, in this study, AS was detected in only 0.03% of the patients before hip replacement surgery. The extremely low incidence of AS might be explained by the following studies: Beydoun et al. found the AS prevalence was higher in those high-income populations. Edwards et al. and Cleveland et al. found the low-income populations have increased potential need for THA [12–14]. Additionally, it may reflect that AS patients were not welcomed by orthopedists or the contribution of AS to mortality and morbidity might have been ignored in the orthopedics department. Unlike low-income countries, high-income countries have already recognized AS as the most important cause of death from VHD [11].

A previous study showed that the near-term risk for pulmonary embolism may be increased by heart diseases not associated with a diagnosed peripheral vein thrombosis [15]. By contrast, VHD acted as the protective factor for pulmonary embolism of the hip replacement in this study. It might because the doctors were fully prepared to deal with such patients. However, further studies are required to determine the detailed protective reason for valvular disease. Some studies have demonstrated that ARF after cardiac surgery is related to insufficient renal blood flow [16]. In this study, among patients with valvular disease, hip replacement was also significantly related to ARF, which may further be related to a low blood flow. In such cases, more attention should be paid to blood creatinine levels of patients with valvular disease before hip arthroplasty, particularly those with renal impairments preoperatively.

Most of the patients with valvular disease are older adults with a poor cardiopulmonary reserve along with several medical comorbidities, such as pneumonia or acute pulmonary edema/failure, as in this study, which means higher cardiopulmonary complications rates followed by higher death rates during the perioperative period. Therefore, lung functions of these older adult patients undergoing hip replacement need to be considered. Furthermore, the anesthesia type needs to be considered. On the one hand, general anesthesia may cause difficulty in extubation or lung infection postoperatively, which may even lead to an increased risk of infection around the prosthesis. However, one study found no relationship between the type of anesthesia and pneumonia after THA [17]. On the other hand, if patients with valvular disease have low blood pressure, local anesthesia is advantageous in reducing the risk of intraoperative hypotension, which may result in further myocardial damage. Moreover, most patients are non-selectively admitted, and a preoperative preparation may be relatively insufficient, leading to an increased risk of perioperative complications.

Compared with valvular disease, AS had fewer postoperative complications. In contrast to other valvular diseases, AS has a significant association with acute hepatic failure. To date, no study has been conducted that clearly finds the progress of acute liver failure (ALF) after hip arthroplasty with valvular disease. Another study had shown that ALF is not caused by hypotension or shock. However, it is related to chronic hemorrhagic heart failure, which is followed by a reduced oxygen supply and portal hypertension. The combined effect results in ALF [18, 19]. Of note, ALF is mainly caused by hepatic hemorrhage rather than low blood output. Nevertheless, hepatic hemorrhage is often associated with advanced VHD. Thus, it may be caused by heart failure, which is the most common end stage of AS, although there may also be other mechanisms awaiting discovery.

Despite some differences between AS and other valvular diseases, they both significantly improve the hospitalization cost and length of stay. Therefore, more attention should be paid to VHD patients undergoing hip replacement.

Our study has several limitations. This study roughly revealed the impact of VHD on hip replacement. The specific effect of various types of valvular diseases other than AS on hip replacement remains to be discussed. In addition, the relatively small size of AS patients provided limited statistical power. Moreover, the severity of the different valvular diseases had been limited by the database.

In conclusion, VHD patients undergoing THA is becoming more common as life expectancy continues to rise. However, the treatment urgency of THA and BHA are unclearly defined, while AS and heart dysfunction are relative contradictions. This study may give a reference to patients with valvular disease undergoing hip replacement in the future and spark future studies regarding the impact of different valvular diseases on hip replacement patient.

## Supplementary Information

**Additional file 1: Supplemental Table 1.** ICD-9 diagnosis codes for postoperative complications.

## Data Availability

The datasets used and/or analysed during the current study are available from the corresponding author on reasonable request.

## Abbreviations

VHD: Valvular heart disease
OR: Odds ratio
AS: Aortic stenosis
HCUP–NIS: Healthcare Cost and Utilization Project – Nationwide Inpatient Sample
