# Supplementary material for: Impact of valvular heart disease on hip replacement: a retrospective nationwide inpatient sample database study
Source: BMC Musculoskelet Disord. 2021 Oct 9;22:860. doi: 10.1186/s12891-021-04738-z (PMC8501620; doi:10.1186/s12891-021-04738-z)
Supplement: Supplementary file 1 — Additional file 1: Supplemental Table 1. ICD-9 diagnosis codes for postoperative complications. [file 12891_2021_4738_MOESM1_ESM.docx]

| **Supplemental Table 1：** ICD-9 diagnosis codes for postoperative complications | |
| --- | --- |
| **Variable** | **ICD-9 Code** |
| **Acute complications** |  |
| ***Surgical complications*** |  |
|  |  |
| Shock | 998.0 |
|  |  |
| Hemorrhage, hematoma, or seroma | 998.1, 998.11, 998.12, 998.13 |
| Accidental perforation or laceration of a blood vessel, nerve, or organ | 998.2 |
| Wound dehiscence | 998.3, 998.30, 998.31, 998.32, 998.33 |
| Foreign body | 998.4 |
| Postoperative infection | 711，7110，71100，71105，7116，71160，7119，71190，71195，730，73000，73005，7301，73010，73015，7302，73025，7309，73090，73095 |
| Fracture of the neck, shaft, or unspecified - femur | 820，8200，82001，82003，82009，8201，82010，82011，82012，82013，82019，8202，82020，82021，82022，8203，82030，8208，8209，8210，82100，82101，8211，82110，82111 |
| Non-healing surgical wound | 998.83 |
| Other unspecified procedural complications | 998.8, 998.81, 998.89, 998.9 |
| DVT/PE | 41511, 41519, 45340, 45341, 45342 |
| Mechanical complication of a prosthetic joint | 99640–99647, 99649 |
| ***Medical complications*** |  |
| Acute cardiac event | 410.0–410.9, 411.1, 411.8, 415.0, 420.0, 420.9, 421.0, 421.1, 421.9, 422.0, 422.9, 427.0–427.5, 428.0–428.9 |
| Acute pulmonary edema/failure | 518.4, 518.81, 518.82, 518.84 |
| Acute cerebrovascular event | 997.00, 997.01, 997.02, 997.09 |
| Acute renal failure | 584.5–584.9 |
| Acute hepatic failure | 570 |
| Pneumonia | 480,480.0, 480.1, 480.2, 480.3, 480.8, 480.9, 481, 482, 482.0, 482.1, 482.3, 482.30, 482.31, 482.32, 482.39, 482.40, 482.41, 482.42, 482.49, 482.8, 482.81, 482.82, 482.83, 482.84, 482.89, 482.9, 483, 483.1, 483.8, 484, 484.1, 484.3, 484.5, 484.6, 484.7, 484.8, 485, 487.0, V12.61, 507.0, 514, 518.4, 518.5, 516, 516.8, 997.31 |
| Sepsis | 995.9, 038.0–038.4, 999.3 |
| Urinary tract infection | 599.0, 996.64, 996.31, V13.02 |
